# Errata

**Published:** 1808-05

**Authors:** 


					ERRATA.
lor 'MCuc-:% .-u.-Ui: F:, running Titles',? for pp. 417?43^ riadpp. 433?44S.

				

